# Programmed Cell Death in the Pathogenesis of Influenza

**DOI:** 10.3390/ijms19072065

**Published:** 2018-07-16

**Authors:** Daisuke Fujikura, Tadaaki Miyazaki

**Affiliations:** 1 Center for Advanced Research and Education (CARE), Asahikawa Medical University, 2-1-1-1 Midorigaoka-Higashi, Asahikawa 078-8510, Japan; 2 Department of Probiotics Immunology, Institute for Genetic Medicine, Hokkaido University, North-15, West-7, Kita-ku, Sapporo 060-0815, Japan

**Keywords:** influenza, pathogenesis, programmed cell death

## Abstract

Influenza is a respiratory disease induced by infection by the influenza virus, which is a member of *Orthomyxoviridae* family. This infectious disease has serious impacts on public health systems and results in considerable mortality and economic costs throughout the world. Based on several experimental studies, massive host immune reaction is associated with the disease severity of influenza. Programmed cell death is typically induced during virus infection as a consequence of host immune reaction to limit virus spread by eliminating niches for virus propagation without causing inflammation. However, in some viral infectious diseases, such as influenza, in the process of immune reaction, aberrant induction of programmed cell death disturbs the maintenance of organ function. Current reports show that there are different types of programmed cell death that vary in terms of molecular mechanisms and/or associations with inflammation. In addition, these novel types of programmed cell death are associated with pathogenesis rather than suppressing virus propagation in the disease course. Here, we review our current understanding of mechanisms of programmed cell death in the pathogenesis of influenza.

## 1. Introduction

The respiratory disease influenza has serious impacts on public health systems and results in considerable economic costs to both individuals and countries. According to a report by World Health Organization (Available online: http://www.who.int/mediacentre/factsheets/fs211/en/, accessed on 31 January 2018) [1], influenza epidemics are estimated to result in millions of severe cases and about 250,000–500,000 deaths each year. In pandemic years such as 1918, when the H1N1 Spanish flu outbreak occurred, the infectious rate reached approximately 5% and the fatality rate was roughly 2% of the world’s population. In the case of the 1968 H3N2 Hong Kong influenza and the 2009 H1N1 pandemic influenza, the global fatality rates were estimated to be approximately 0.2%.

The causative agents of the disease are strains of the influenza virus, which is a member of the *Orthomyxoviridae* family. The genome of the virus consists of single-stranded, segmented, negative sense RNA. Influenza viruses are divided into three types, A, B and C, based on the antigenic differences among their matrix protein, M1, and nucleic protein, NP. Each of the three virus types is also distinguished by their serological type of viral surface molecules such as hemagglutinin (HA) and neuraminidase (NA) [2].

The influenza virus can be spread as an aerosol from coughing or sneezing by the infected patient. It can also be spread by touching surfaces contaminated by the virus. The symptoms of the disease are nasal inflammation, high body temperature (over 100.4 °F), aching muscles, chills and sweats, headache, persistent cough, fatigue and weakness. The disease often induces a serious respiratory tract disorder, such as severe pneumonia. Because of its high pathogenicity, damaging impacts on public health and economic costs, in this review, we focus on type A seasonal influenza virus.

Programmed cell death (PCD) is the deliberate suicide mechanism that removes unwanted cells, such as tumors or autoreactive lymphocytes, from the body to maintain homeostasis. In contrast to necrosis, which is a form of cell death that results from accidental tissue injury and provokes an inflammatory response, PCD is carried out without inflammation. During virus infection, the infected cells induce PCD as a consequence of activation of host cellular defense mechanism to limit the virus spread by removing the infected cells. Currently, increasing evidence suggests an association of PCD with suppression of virus replication as well as regulation of inflammation and pathogenicity of the influenza disease.

Usually, dead cells brought about by PCD are eliminated by a phagocytotic process of tissue resident macrophages because the dead cells should be source of auto-antigens that lead to autoinflammation and an autoimmune response [3]. Therefore, dead cells are hardly detectable on tissue slides obtained from the lung of healthy donors. However, in the case of severe influenza virus infection, dead cells can be observed on the airways and alveoli of the lungs of infected donors. Influenza virus targets mainly airway and alveolar epithelial cells in vivo [4,5]. Rapid replication of the virus in these cells disturbs the cellular function and damages tissue as well as causing production of a huge number of dead cells in the lung of the infected host. The rapid accumulation of dead cells should disturb phagocytotic function of lung resident macrophages. Influenza viruses infect and also disturb alveolar macrophages that phagocyte pathogens and maintain lung functions such as gas exchange [6]. It is also reported that anti-influenza virus immune-complex suppresses phagocytic function of lung-resident macrophages [7].

Currently, PCD is broadly classified into three types, apoptosis, necroptosis and pyroptosis, based on the process inducing the type of PCD. Among the PCD types, some types are associated with the regulation of host immune protection and tissue inflammation. In this review, we focus on the details of the molecular mechanisms that induce PCD and the association of each type of PCD with pathogenesis of influenza.

## 2. Infection and Virus Replication

Among the three types A, B and C, influenza viruses have closely related structures. The genome of the virus consists of seven or eight segments of negative-sense RNA. Each segment encodes one or more genes. The influenza A virus genome consists of eight segments. These segments contain several genes: HA, NA, nucleoprotein (NP), matrix protein (M)1, M2, non-structural (NS)1, NS2, polymerase acidic protein (PA), polymerase basic (PB)1, PB1-F2 and PB2 [8]. The indentification of eight novel open reading frames has been reported: PB1-N40, PB2, PB2-S1, PA-X, PA-N155, PA-N182, M42, and NS3 [9].

Influenza virus infection and replication consist of a multi-step process [10]. The virus particles contain the glycoproteins HA and NA on the viral surface. HA targets and binds to sialic acid of glycosylated protein on the cell surface of host epithelial cells, typically in the nose, throat, and lungs of mammals [11]. The cell imports the virus by endocytosis and then the HA is cleaved by a protease in the host cellular endosome. In the endosome, the acidic conditions cause fusion of the viral envelope with the host endosomatic membrane. In this stage, the viral M2 protein acts as a proton channel to acidify the core of the virus particle. The acidification of the virus particle causes it to disassemble and release the viral ribonucleo protein (vRNP) that consists of genomic negative sense RNA (vgRNA) and core proteins, such as accessory proteins and RNA-dependent RNA polymerase, into the host cell cytosol. In the host cell cytosol, the vRNPs are transported into the cell nucleus. In the nucleus, the viral RNA polymerase transcribes premature viral messenger positive sense RNA (vmRNA) as well as complementary positive-sense RNA (cRNA) that acts as a template for newly synthesized vgRNA. The new vRNPs and vmRNAs are exported into the cytoplasm. Viral HA and NA proteins translated from the mature vmRNA are secreted through the Golgi apparatus onto the cell surface. Synthesized viral polymerases and accessory proteins are transported back into the nucleus to bind vgRNA and form new vRNP. Other viral proteins play roles such as degrading host cellular mRNA and using the released nucleotides for vmRNAs synthesis, and inhibiting translation of host cellular mRNAs in host cell cytosol. Synthesized HA and NA proteins cluster and form membrane protrusion on the host cell membrane. The protrusion incorporates the negative strand vRNA and viral core proteins left from the host nucleus to form a mature virus particle. In this stage, the mature virus particle adheres to the cell through HA. Viral NA can cleave the sialic acid residues of host cell surface proteins, to release the particle from the cells.

## 3. Cytokine Dependent Induction of Programmed Cell Death (PCD) in Influenza Virus Infection

**Figure 1:**
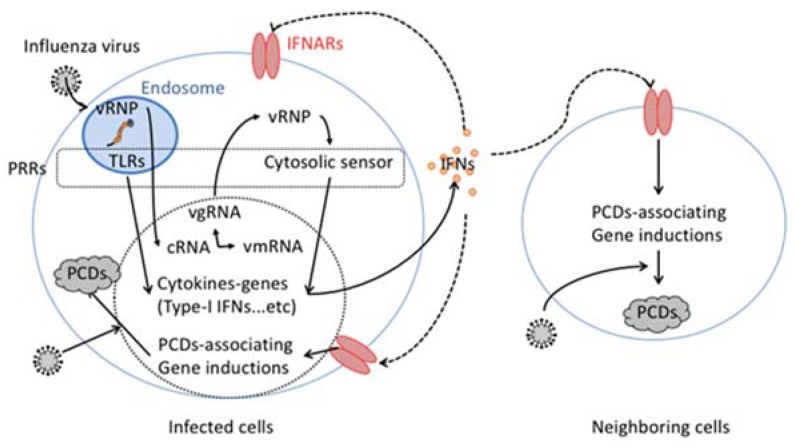
Cytokine-dependent pogrammed cell death (PCD) induction in influenza virus infection. VRNP incorporated in influenza virus particles is released from the particle within the host cellular endosome. The released vgRNA that consists of vRNP engages host endosomatic pattern recognition receptors (PRRs), such as toll like receptors (TLRs). In the host cell cytosol, vRNP also activates cytosolic PRRs. The activation of both endosomatic and cytosolic PRRs stimulate gene induction of type-I interferon (IFN). The secreted type-I IFN binds and activates its specific receptor, IFNARs in an autocrine and paracrine manner. The activated IFNARs stimulate the gene induction of several PCD associated genes, including FasL or TRAIL.

In response to influenza virus infection and replication, the induction of PCD is triggered in a host cytokine-dependent manner (Figure 1) [12,13]. The influenza virus particles bind to sialic acid on the host cell surface via their surface molecules HA and NA. In the influenza virus infection stage, the viral particles are enzymatically resolved in the endosome of the cells. At this stage, vgRNAs released from the viral particles stimulate pattern recognition receptors (PRRs) including toll like receptors (TLRs), which localize on the endosomal plasma membrane [14,15]. The stimulated TLRs activate intracellular signals to induce gene expression of several cytokines and intracellular molecules.

Some virus particles, having escaped host enzymatic degradation, fuse endosomatic plasma membrane and inject vRNP. The other intracellular PRRs including retinoic acid-inducible gene-I (RIG-I) like molecules, Z-DNA-binding protein 1(ZBP1)/DNA-dependent activator of IFN-regulatory factors (DAI), nucleotide-binding oligomerization domain (NOD)-like molecules, or absent in melanoma 2 (AIM2)-like molecules should recognize the injected and/or de novo vRNP in cytozol to activate intracellular signals promoting several cytokine gene inductions [16]. Among cytokines produced by PRRs activation, type-I IFNs are rapidly and abundantly produced to interfere with virus spread. By binding to the specific receptors (IFNARs), the type-I IFNs stimulate several gene inductions in infected cells as well as neighboring cells in an autocrine and paracrine manner. Among type-I IFNs-stimulating genes (ISGs), intracellular enzymes, including RNase L and 2′-5′-oligoadenylate synthetase 1 (2′-5′-OAS1), degrade viral RNAs to stop production of virus components in the infected cells [17,18]. Type-I IFNs also induce expression of PCD-associated molecules including receptors, ligands and intracellular signaling molecules to destroy niches in which the virus can propagate [13,19]. Several papers report that some influenza virus proteins such as NS1, PB1-F2 and PA disturb this signaling associated with IFNs [20,21,22,23,24,25,26]. Therefore, type-I IFNs effectively shut down virus propagation by sequentially activating intracellular enzymatic antiviral mechanisms as well as extracellular antiviral cell death-inducing systems. Because of their effective replication in host cells, viruses also have mechanisms to interpret the host antivirus mechanism mediated by type-I IFN [27]. In addition, accumulating evidence indicates the side effect of type-I IFN signal in several infectious and noninfectious diseases [13,28].

## 4. Apoptosis in the Pathogenesis of Influenza

Apoptosis is the most common form of PCD. When cells induce apoptosis, they shrink their cellular shapes and involve their intracellular components (including viral components, when infected with viruses) into their cytoplasm to prevent unwanted immune responses.

**Figure 2:**
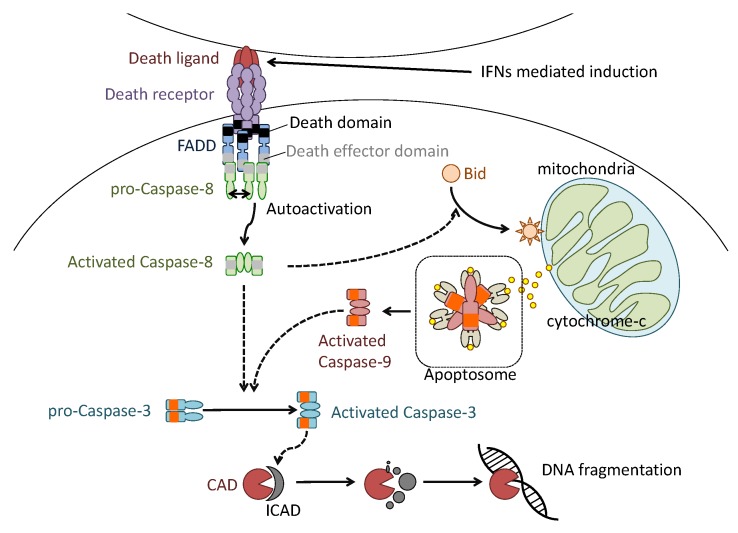
Molecular defined apoptosis induction in influenza virus infection. Type-I IFN-mediated death ligand expression on the host cell surface aggregates death receptor (DR) trimerization. DR trimerization induces aggregation of intracellular adaptor proteins such as Fas-associated protein with death domain (FADD), which binds the cytoplasmic tail of DR. The FADD aggregation also induces pro-caspase-8 aggregation and enzymatic autocatalytic activation. The activated caspase-8 cleaves and activates effector caspase-3 and intracellular BH3 interacting domain death agonist protein (Bid). The activated Bid induces permeabilization of the mitochondrial outer membrane. The permeabilization induces the release of cytochrome-C from the interior of mitochondria. The released cytochrome-C binds caspase-9 and forms apoptosome. The formation of apoptosome induces aggregation and auto-enzymatic activation of caspase-9 to activate caspase-3. The caspase 3 activated by activated caspases 8 and 9 cleaves and inactivates ICAD to induce fragmentation of genomic DNA of cells.

The intracellular mechanism of apoptosis induction is molecularly defined (Figure 2). Most apoptotic induction depends on an intracellular cascade consisting of catalytic activation of cysteine-dependent aspartate-directed protease (caspases). Caspases are divided into three main groups: initiator caspases (caspases 2, 8 and 9), effector caspases (caspases 3, 6 and 7) and inflammatory caspases (human caspases 1, 4 and 5; and mouse caspases 1 and 11). Initiator caspases activate through homophilic dimerization, resulting in the autocatalytic cleavage. The activated initiator caspases cleave and activate effector caspases. The activated effector caspases lead to activation of caspase-activated DNase (CAD) for fragmentations of genomic DNA of the cells by destroying inhibitor of CAD (ICAD) [29]. The effector caspases also affect formation of cytoskeletal proteins including actin [30].

During virus infection, activation of initiator caspases is triggered by several cytokines. These cytokines are called death ligands, which are type-II transmembrane proteins that include tumor necrosis factor (TNF), Fas ligand (FasL) or TNF-related apoptosis-inducing ligand (TRAIL). In the naïve state, death ligands present as homophilic trimers, whereas the specific receptors (death receptor, DR) present as monomers on cell surfaces. The trimeric complex of death ligands engages monomeric DRs to induce aggregation of the receptors and adaptor proteins that bind to cytoplasmic tail of the receptors. Among the adaptor proteins, Fas-associated protein with death domain (FADD) recruits pro-caspase-8 to its cytoplasmic tail and induces homophilic activation of caspase-8. Activated caspase-8 cleaves and activates its downstream caspases such as effector caspase-3. Caspase-8 also cleaves pro-apoptotic B-cell lymphoma 2 (Bcl2) family proteins, such as BH3 interacting domain death agonist protein (Bid). The cleaved and activated Bid mediates mitochondrial damage and the loss of mitochondrial membrane potential to release cytochrome-c to the cytosol from the interior of mitochondria. The released cytochrome-c binds to apoptotic protease activating factor 1 (Apaf1), an adaptor protein, to form “Apoptosome”, a protein complex that induces auto activation of initiator caspase-9. The activated caspase-9 also cleaves and activates caspase-3 to amplify the apoptotic signal cycle.

During influenza A virus infection, functional defects on FasL, a specific ligand of Fas receptor, protect mice. However, deficiency of the FasL gene has almost no effect on virus propagation in mouse lung during lethal influenza virus infection [31,32]. During lethal infection with the virus, expression of FasL is abundantly observed on several cell types of the lung, such as epithelial cells, macrophages, dendritic cells and lymphocytes. The time course of FasL gene expression in the lung of virus-infected mice correlates with that of body weight loss in mice during the lethal virus infection [31]. During the lethal virus infection, aberrant production of type-I IFN is also observed. Type-I IFN production is critical to the induction of FasL expression because functional deficiency of the type-I IFN signal prevents FasL expression on the cell surface of several cells that consist of lung in mice lethally infected with influenza virus. In addition, administration of anti-Fas agonistic antibody induces acute lung inflammation in an uninfected condition [33,34]. Collectively, FasL-mediated apoptosis is associated with severity of illness such as host body weight loss, lung inflammation and reduced survival rate but not with suppression of virus replication in severe influenza virus infection.

Induction of TRAIL expression is also linked to influenza pathogenicity. In contrast to FasL, the role of TRAIL is more complex in protecting the host during influenza virus infection. Functional defects in the TRAIL gene in mice cause reduced survival rate during mild influenza virus infection [35]. However, anti-TRAIL neutralizing antibody protects mice and reduces apoptosis induction in airway epithelial cells and alveolar leakage during severe or lethal influenza virus infection [36,37,38]. Unlike FasL expression, TRAIL expression is limited on the infected epithelial cells, virus specific CD8 T cells, dendritic cells and macrophages during influenza virus infection. A functional defect of TRAIL causes increased propagation of the virus [35]. Therefore, TRAIL is crucial for the suppression of infection and propagation of influenza virus and the significance of TRAIL on host survival depends on the severity of the disease symptoms after the virus infection.

## 5. Necroptosis in the Pathogenesis of Influenza

Necrosis is often considered as an accidental and unregulated cellular event. However, increasing reports indicate that the phenomenon is also induced by the programmed mechanism in response to several external stimulations such as virus infection. This programmed necrosis is called necroptosis. Necroptosis is an alternative cell death program and is histologically distinguished from apoptosis. When cells induce necroptosis, the cell body bursts and immediately releases intracellular components (called as damage-associated molecular patterns, DAMPs) that can trigger inflammatory reaction mediated by the innate immune system. The molecular mechanisms that induce necroptosis and their differences compared with those of apoptosis are currently discussed [39,40].

**Figure 3:**
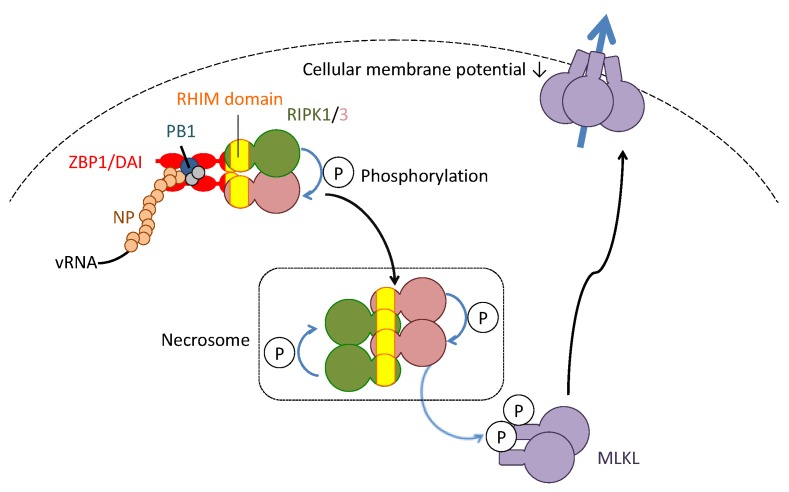
Molecularly-defined necroptosis induction in influenza virus infection. The complex of vRNA and nucleoprotein (NP) and polymerase basic protein 1 (PB1) of the influenza virus is recognized by the Z-DNA-binding protein 1/DNA-dependent activator of IFN-regulatory factors (ZBP1/DAI). The ZBP1/DAI induces oligomerization of receptor-interacting kinase (RIPK)1/3. Within the oligomeric kinase complex (namely, the necrosome), each RIPK phosphorylates and activates together. The activated RIPKs phosphorylate and activate mixed lineage kinase domain-like (MLKL). The phosphorylated MLKL translocates from the cytosol to the cellular membrane. The translocated MLKLs form a cellular channel to permeabilize the cellular membrane.

The induction of necroptosis depends on the formation of necrosomes that consist of at least three kinases: receptor interacting kinase (RIPK)-1 and -3 and mixed lineage kinase domain-like (MLKL) (Figure 3). The interaction between RIPK1 and RIPK3 depends on RIP homotypic interaction motif (RHIM), which is conserved among these molecules. In the process of the necrosome formation, both RIPK1 and RIPK3 reciprocally phosphorylate and activate together, resulting in phosphorylation of MLKL. The phosphorylated MLKL translocates to cell membranes where MLKL binds to phosphatidylinositol phosphates to induce permeabilization of the cell membrane and depolarization of the cellular membrane potential.

Necroptosis is triggered by several pro-apoptotic stimuli including the death ligands, toll-like receptor agonists or genotoxic stress when caspase activity is blocked [41]. Therefore, necroptosis seems to compensate for the function of apoptosis to remove unwanted cells, such as tumors that are often resistant to apoptotic induction, with inflammation [42]. However, necroptosis has a unique feature in host protection against virus infection. During influenza virus infection, necroptosis is induced by one of the PRRs, ZBP1/DAI, which contains RHIM within its amino-terminal region [43,44]. ZBP1/DAI can bind to NP and polymerase subunit, PB1 of the influenza A virus specifically by its carboxyl terminal region to activate necrosomes containing RIPK3.

The role of necroptosis in host protection against the influenza virus infection is highly controversial. ZBP1/DAI deficiency protects mice during lethal influenza virus infection, although the virus titer is higher in ZBP1/DAI deficient than in wild-type mice [43]. Another study reported that ZBP1/DAI deficiency leads to a high mortality rate of mice infected with influenza virus [45]. In in vitro experiments, ZBP1/DAI-deficient cells induce almost no necroptosis during virus infection [43,45]. Under in vitro culturing conditions, ZBP1/DAI-deficient cells also produce almost no proinflammatory cytokines such as IL-1β, IL-18, IL-6 and TNFα in response to the infection [43].

Based on studies using mice with defects of other necroptosis-related molecules, RIPK3 deficiency leads a high mortality rate in mice infected with influenza virus [44,45,46]. RIPK3 deficiency also reduces production of type I IFN and promotes excessive inflammation including lymphocyte accumulation in the lungs of infected mice. Virus titers in the lungs were sustained in RIPK3-deficient mice compared with wild-type mice. In vitro experiments showed that RIPK3 deficiency protects cells from lysis during influenza virus infection. [44,46]. Because MLKL deficiency does not affect the survival rate of mice during influenza virus infection, the increased susceptibility observed in RIPK3 deficient mice is independent of necroptosis induction.

Taken together, these studies suggest the importance of necroptosis in regulating influenza virus-mediated cell death in in vitro experiments. DAI-mediated necroptosis also has a crucial role in suppressing virus propagation in vivo.

## 6. Pyroptosis in the Pathogenesis of Influenza

**Figure 4:**
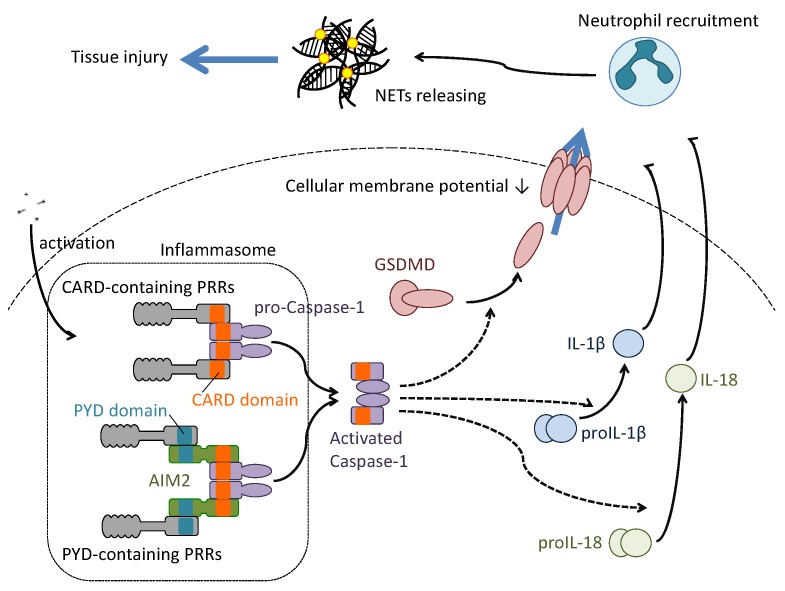
Molecularly-defined pyroptosis induction in influenza virus infection. Several components of the influenza virus particle are recognized by both CARD-containing and PYD-containing PRRs. Recognition by the PRRs induces aggregation of caspase-1 to activate the cellular enzyme. The aggregation of caspase 1 by PYD-containing PRRs needs the adaptor protein absent in melanoma 2 (AIM2), which contains the PYD and CARD domains on its amino acid structure. The activated caspase 1 cleaves gasdermin D (GADMD), proIL1β or proIL18 to convert these substrates to the active form. The activated GSDMD translocates from the cytosol to the cellular membrane to form a channel. The formation of the channel induces permeabilization of the cellular membrane to release IL1β and IL18. These cytokines, once released, stimulate and recruit neutrophils to the infected local area. The recruited neutrophils endocytose the virus particles and release neutrophil extracellular traps (NET) to capture the pathogens. However, the host tissue damage brought about by the abundant production of NETs should be considered.

Pyroptosis is a type of cell death that is associated with production of pro-inflammatory cytokines including IL-1β and IL-18 [47,48]. The intracellular mechanism of pyroptosis induction depends on formation of inflammasomes, which are protein complexes that activate inflammatory caspases including caspase-1 (Figure 4). Similar to necroptosis, pyroptotic cells reduce a potential on the plasma membrane and burst the cells. The pyroptotic cells also exhibit apoptotic characteristics, such as caspase-dependent mechanism, chromatin condensation and DNA fragmentation.

In cells infected with intracellular microbial pathogens, several microbial components are recognized by cytosolic PRRs, which conserve a caspase activation and recruitment domain (CARD) or a pyrin domain (PYD), such as NOD-like receptor proteins (NLRPs), absent in melanoma (AIM) and pyrin. The CARD-containing PRRs (NLRP1 and NLRC4) directly recruit pro-caspase-1 to their CARDs for aggregation and form inflammasomes to activate caspase-1. Recruitment of apoptosis-associated speck-like protein containing CARD (ASC) is required for formation of inflammasome of PYD-containing PRRs (neuronal apoptosis inhibitory protein (NAIP), MHC class II transcription activator (CIITA), incompatibility locus protein from *Podospora anserina* (HET-E) and telomerase-associated protein (TP1) (NACHT), leucine-rich repeats (LRR) and PYD domains-containing protein 3 (NLRP3)) and PRR-adaptor protein to take place. The activated caspase-1 cleaves and converts pro-IL-1β and pro-IL-18 into their mature forms.

Caspase 1 deficiency reduces the survival rate of mice during non-lethal challenge with influenza virus [49], whereas the caspase-1 deficiency protects mice against lethal infection of the virus [50]. Inflammasome formation occurs within the infected stromal and hematopoietic compartment in mice infected with the influenza virus. In addition, the inflammasome activation in the hematopoietic compartment is necessary to protect mice [49]. A recent study using fluorescent reporter mice that visualizes formation of inflammasome in vivo showed that, in the early phase of virus infection, inflammasome formation is limited in the virus-infected lung stromal cells, and that inflammasome formation in the hematopoietic compartment occurs in the late phase of the infection [51]. Based on these studies, the role of the inflammasome in host protection depends on cell type or infectious stage after the virus infection.

Activation of caspase-1 in the hematopoietic but not the stromal compartment recruits several inflammatory leukocytes to the lung during virus infection. These include macrophages, dendritic cells, natural killer cells, neutrophils, and lymphocytes. Recruitment of the cells contributes to the induction of host antiviral immunity [49] as well as to the inflammatory process during lethal influenza virus infection [50,52]. Among the cells recruited in the local microbial inflammation area, neutrophils phagocytose microbes and produce reactive oxygen species, whose accumulation often leads to tissue damage. The neutrophils also produce neutrophil extracellular traps (NETs), a network of extracellular fibers that consist of their own genomic DNA and some proteases including elastase and cathepsin G to prevent further spread of the pathogens [53]. Despite the beneficial effects of NETs, accumulation of NETs is also associated with the inflammatory process in several diseases [54]. During lethal influenza virus infection, massive accumulation of neutrophils leads to excessive production of NETs, thus causing acute lung injury. In this situation, macrophages reduce the accumulation of NETs—and the spread of inflammation via their phagocytotic role [52].

## 7. Conclusions

**Figure 5:**
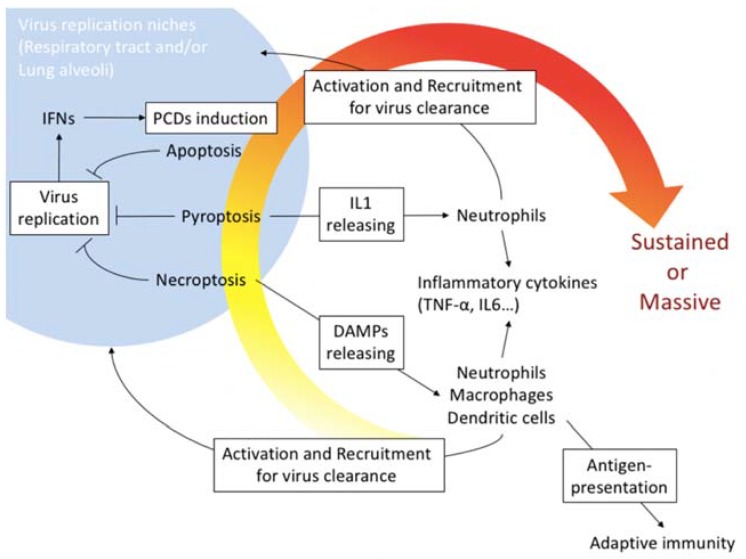
PCD induction in host protective immunity and pathogenicity in influenza virus infection. PCDs including apoptosis, necroptosis and pyroptosis are usually mechanisms to limit influenza virus proliferation niches. Especially, necroptosis and pyroptosis associate releasing DAMPs or IL1 that activate and recruit phagocytotic cells such as neutrophils, macrophages and dendritic cells into local virus replication area. The phagocytotic cells produce several inflammatory cytokines including TNFα and IL-6 to promote the accumulation of the cells. High yield of the virus replication often promotes and/or sustains the massive host immunoreactions that cause tissue damages and organ failure.

In this review, we discuss the molecular mechanisms of the three main types of PCD, and their association with the pathogenicity of the seasonal influenza virus infection in mammals (Figure 5). As described above, the role of PCD in host survival exhibits the opposite effect in non-lethal challenge with the virus compared with lethal challenge of the virus. The immune reaction is basically the mechanism to protect host. However, the aberrant regulation on the mechanism often leads tissue damages in some situations such as auto immunological disorders. In the case of influenza virus infection, induction of PCD depends on the production of viral RNAs, which is a consequence of massive and rapid replication of the influenza virus. PCD is a cellular suicide mechanism. It means that the induction effectively removes host cells infected with the virus. However, the cells have important roles in maintaining the homeostasis of the host body when they are not infected. Therefore, understanding what cell type is infected with the virus is important in considering the role of PCD in host survival from the disease [55]. From this viewpoint, tissue tropism or replication frequency of highly pathogenic avian influenza viruses such as H5N1 type virus have some differences when compared with those of seasonal influenza virus in mammals [11,56,57,58,59].

To control influenza, the first choice for prevention is immunization with influenza vaccines, which induce virus-specific antibodies and cellular responses to eliminate the viruses. These responses depend on antigenicity of the virus, which reduces the effectiveness of this strategy for completely preventing the spread of the virus. This is because the viruses can easily change their antigenicity in response to host antigenic pressures. The genetic diversity of the virus is derived from its RNA-dependent RNA polymerase, which lacks a proofreading system, which DNA polymerase has. This characteristic of the influenza virus (referred to as “antigenic shift”) accumulates point mutations on their genomic sequences and facilitates their escape from host immunological reactions, which consist of antiviral protein-specific antibodies and cytotoxic T lymphocytes. The influenza virus also has another characteristic, which leads its escape from host immune reactions. The viral segment can easily transmit among the different subtypes when they co-infect within the same cell (referred to as “antigenic drift”). This phenomenon can result in rapid and frequent change of the virus serotype [2].

Currently, for therapeutic treatment, several anti-influenza virus reagents such as amantadine have been developed and are used clinically. These reagents are antagonists that selectively block the catalytic pocket of viral proteins, and their specificity depends on the structure of the targeted viral protein [10]. Therefore, the virus can also escape from the pressure of these antiviral reagents because the influenza virus can easily change its protein structures via antigenic drift and antigenic shift. In fact, some amantadine-resistant subtypes of the virus have already been reported [60]. Antiviral protein therapies including vaccinations and chemical drugs are actually effective; however, there are problems depending on the unique characteristics of the influenza virus, such as antigenic drift and antigenic shift. The mechanisms of PCDs are usually quite complex and modified by several factors such as the presence of microparticles, e.g., exosomes [61,62] or virus specific proteins. The mechanisms basically depend on host molecules, as described above. In addition, computational models that based on the PCD mechanism are often employed to better understand cause–effect relationships and to make qualitative and quantitative predictions relevant to clinical conditions [63,64,65]. Several proteomics studies provide information on host molecules that are important for virus replication [66]. A better understanding of the molecular mechanisms of PCD induction could provide us novel approaches to combat influenza.

## Abbreviations

HA	Hemagglutinin
NA	Neuraminidase
PCD	Programmed cell death
NP	Nucleoprotein
M	Matrix protein
NS	Non-structural protein
PA	Polymerase acidic protein
PB	Polymerase basic protein
M	Matrix protein
vRNP	Viral ribonucleoprotein
vgRNA	Viral genomic RNA
vmRNA	Viral messenger positive sense RNA
cRNA	Complementary positive-sense RNA
PRR	Pattern recognition receptors
TLR	Toll like receptor
RIG-I	Retinoic acid-inducible gene-I
ZBP1/DAI	Z-DNA-binding protein 1/ DNA-dependent activator of IFN-regulatory factors
NOD	Nucleotide-binding oligomerization domain
AIM2	Absent in melanoma 2
IFN	Interferon
ISGs	Interferon stimulated genes
2′-5′-OAS	2′-5′-oligoadenylate synthetase 1
CAD	Caspase-dependent Dnase
ICAD	Inhibitor of CAD
TNF	Tumor necrosis factor
FasL	Fas ligand
TRAIL	TNF-related apoptosis-inducing ligand
DR	Death receptor
FADD	Fas associated protein with death domain
Bcl2	B-cell lymphoma 2
Bid	BH3 interacting domain death agonist protein
Apaf-1	Apoptotic protease activating factor 1
DAMPs	Damage-associated molecular patterns
RIPK	Receptor interacting kinase
MLKL	Mixed lineage kinase domain-like
RHIM	RIP homophtypic interaction motifs
IL	Interleukin
CARD	Caspase activation and recruitment domain
PYD	Pyrin domain
NAIP	Neuronal apoptosis inhibitory protein
CIITA	MHC class II transcription activator
HET-E	Incompatibility locus protein from *Podospora anserina*
TP1	Telomerase-associated protein
NACHT	NAIP, CIITA, HET-E and TP1
LRR	Leucine-rich repeats
NLRP	NACHT, LRR and PYD domains-containing protein
ASC	Apoptosis-associated speck-like protein containing CARD
NETs	Networks of extracellular fibers
